# Supplementary material for: Coupled power generators require stability buffers in addition to inertia
Source: Sci Rep. 2022 Aug 12;12:13714. doi: 10.1038/s41598-022-17065-7 (PMC9374790; doi:10.1038/s41598-022-17065-7)
Supplement: Supplementary file 1 — Supplementary Information. [file 41598_2022_17065_MOESM1_ESM.pdf]

Supplementary Materials for

# Coupled power generators require stability buffers in addition to inertia

Gurupraanesh Raman†, Gururaghav Raman†, and Jimmy Chih-Hsien Peng*

* Corresponding author. E-mail: jpeng@nus.edu.sg
† Equally contributing authors

This document is structured as follows:

- **Section S1** (*page 2*) shows how the effectiveness of contributing stability storage varies with system parameters such as the number of virtual synchronous generators, system inertia, and system non-synchronous penetration;
- **Section S2** (*page 4*) describes the Greater London distribution network used in this study and its parameters;
- **Section S3** (*page 6*) presents the control block diagram for the inverters used in the study;
- **Section S4** (*page 7*) describes how we performed the stability analyses for grids with rotating and virtual synchronous generators;
- **Section S5** (*page 8*) explains how the distributed stability metric (DSM) was derived.

# S1 Supplementary note 1: Effectiveness of stability storage for varying system parameters

The results shown in Fig. 5 in the main article correspond to a scenario with 30 virtual synchronous generators (VSGs) in the Greater London power grid, with varying values of virtual impedance contributed by each VSG. This section presents how the effectiveness of contributing stability storage varies when (i) the number of VSGs in the network varies as 10, 30, and 50; (ii) the overall system inertia constant varies as 4 s, 6 s, and 8 s; and (iii) the system non-synchronous penetration (SNSP) varies as 25%, 50%, and 75%. These results are presented below in Fig. S1.

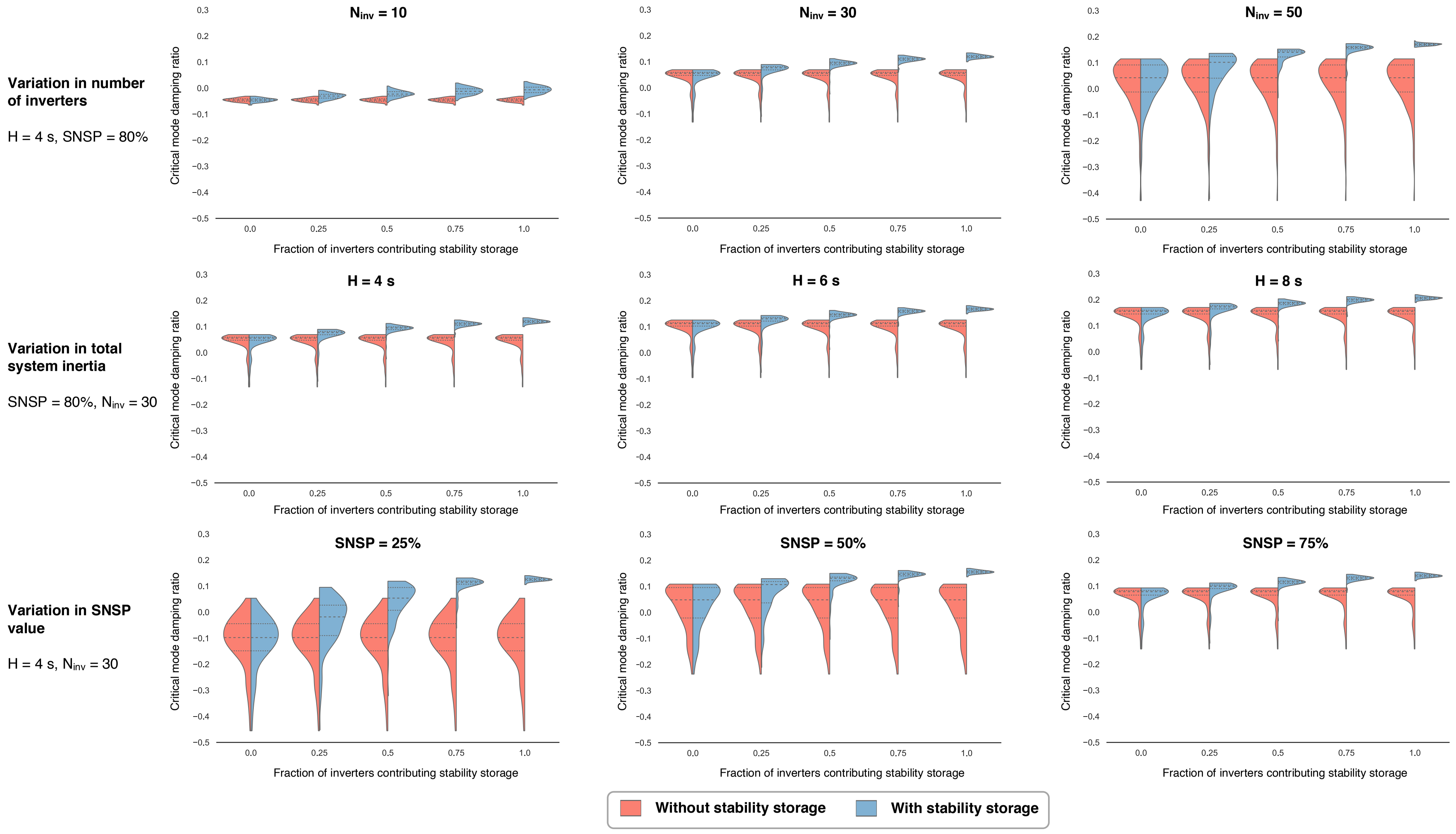


**Fig. S1.** How the effectiveness of contributing stability storage to the power grid (see Fig. 5 in the main article) varies when the number of inverters in the network $N_{inv}$, system inertia constant $H$, and SNSP vary. In all cases, the inverters contribute a fixed virtual impedance of 10 mH.

## S2 Supplementary note 2: Grid parameters for the Greater London distribution network

The network topology for the Greater London power distribution system was obtained using the algorithm presented in ref. [1], and is based on the assumption that power lines are generally laid alongside the road network in a city. The network is shown below in Fig. S2, consisting of nine interconnected subnetworks. Each of the subnetworks comprises a spanning tree of distribution lines that is fed by a 400kV/230kV substation located at its root node. This topology is available for download from ref. [2].

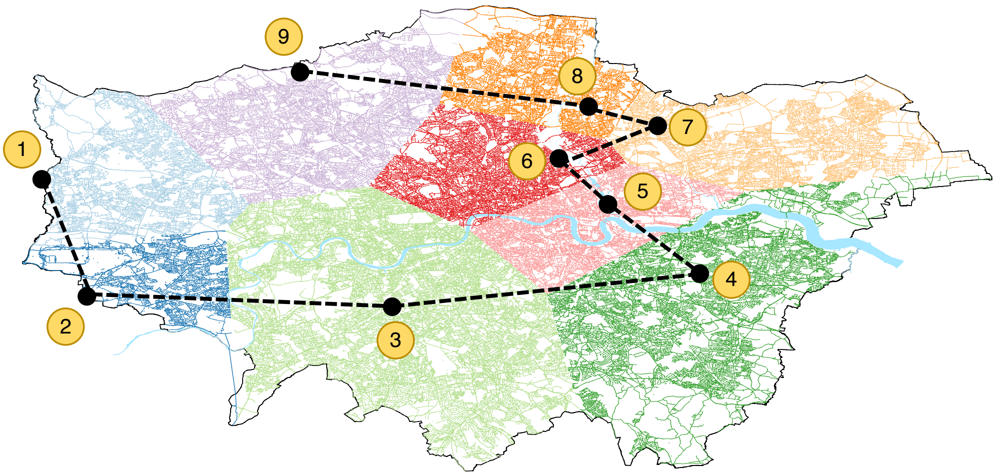


**Fig. S2.** Power distribution network of the Greater London area used in this study. The locations of the substations feeding each of the 9 subnetworks and the lines interconnecting them are also depicted in the figure.

### S2.1 Line parameters

We selected the line impedance values for the grid based on publicly available information about their typical resistance/reactance (R/X) ratio and impedance per length.

*R/X ratio*: As specified in Table 23 of IEEE Standard C37.010-2016 [3], the X/R ratio of the interconnecting lines for the voltages considered in our study are typically in the range of 2-16 for open wire lines, and 1-3 for underground cables. Since the above ranges are specified for a power frequency of 60 Hz, they are converted to the respective ranges 1.67–13.33 and 0.83–2.50 for 50 Hz, which is the case for our study. The overall range 0.83–13.33 for X/R translates to 0.08–1.20 for R/X. Here, it is pertinent to note that the distribution lines in each of the 9 subnetworks are classified into 8 different tiers, with the lines closest to the substation or laid alongside larger roadways assigned as Tier-0, and the ones serving the leaf nodes or laid alongside smaller roads assigned as Tier-7; see ref. [1] for more details. The lines interconnecting the substations are assumed to be Tier-0 and 20 km long. We assign to each tier of lines an R/X ratio in the above range; see Table S1.

*Impedance per length*: The resistance per length for each tier is assigned based on data pertaining to commercially available conductors [4]; see Table S1. These values are in accordance with the UK standard BS5467 for LV cables [5] and BS6622 for MV cables [6]. The impedance per km values so obtained are divided by the appropriate base impedance values to convert them into per-unit (p.u.) values.

Finally, for each line in the network, its impedance is obtained as follows:

$$\text{Line resistance (p.u. }\Omega) = \text{Resistance per length (p.u. }\Omega/\text{km}) \times \text{Length (km)}, \tag{1}$$

and

$$\text{Line reactance (p.u. }\Omega) = \text{Resistance per length (p.u. }\Omega/\text{km}) \times \text{Length (km)} \times \frac{1}{R/X}. \tag{2}$$

**Table S1.** Parameters for the different distribution line tiers. Base power: 10 GVA.

| Tier | Base voltage (kV) | R/X | Resistance per length (Ω/km) | Base impedance (Ω) | Per-unit resistance per length (p.u. Ω/km) |
|---|---|---|---|---|---|
| 0 | 230 | 0.6 | 0.10 | 5.2900 | 1.8904e-2 |
| 1 | 132 | 0.7 | 0.15 | 1.7424 | 8.6088e-2 |
| 2 | 132 | 0.7 | 0.15 | 1.7424 | 8.6088e-2 |
| 3 | 33 | 0.8 | 0.20 | 0.1089 | 1.8365 |
| 4 | 33 | 0.8 | 0.20 | 0.1089 | 1.8365 |
| 5 | 11 | 1.0 | 0.25 | 0.0121 | 20.6610 |
| 6 | 11 | 1.0 | 0.25 | 0.0121 | 20.6610 |
| 7 | 0.4 | 1.2 | 0.30 | 1.600e-5 | 1.8750e4 |

# S3 Supplementary note 3: Control block diagram for virtual synchronous generator

This section presents the control scheme for the virtual synchronous generators (VSG) used in this study. The overall control block diagram is shown in Fig. S3. The current and voltage measurements obtained at the inverter terminals are converted to equivalent DC quantities along the direct ($d$) and quadrature ($q$) axes using the Park's transformation, whereby PI controllers can be used to regulate them as desired. The references for the inverter's output voltage and frequency are determined by the VSG control loop to emulate inertia and enable automatic power sharing amongst multiple such inverters.

The VSG voltage reference $V_{droop}$ is augmented by an emulated voltage drop dependent on the desired output impedance $L_{out}$. The thus-obtained voltage references $V_{ref,dq}$ are tracked through cascaded voltage and current loops whose bandwidths are designed to be significantly faster than that of the VSG controller to avoid any dynamic interaction between each other.

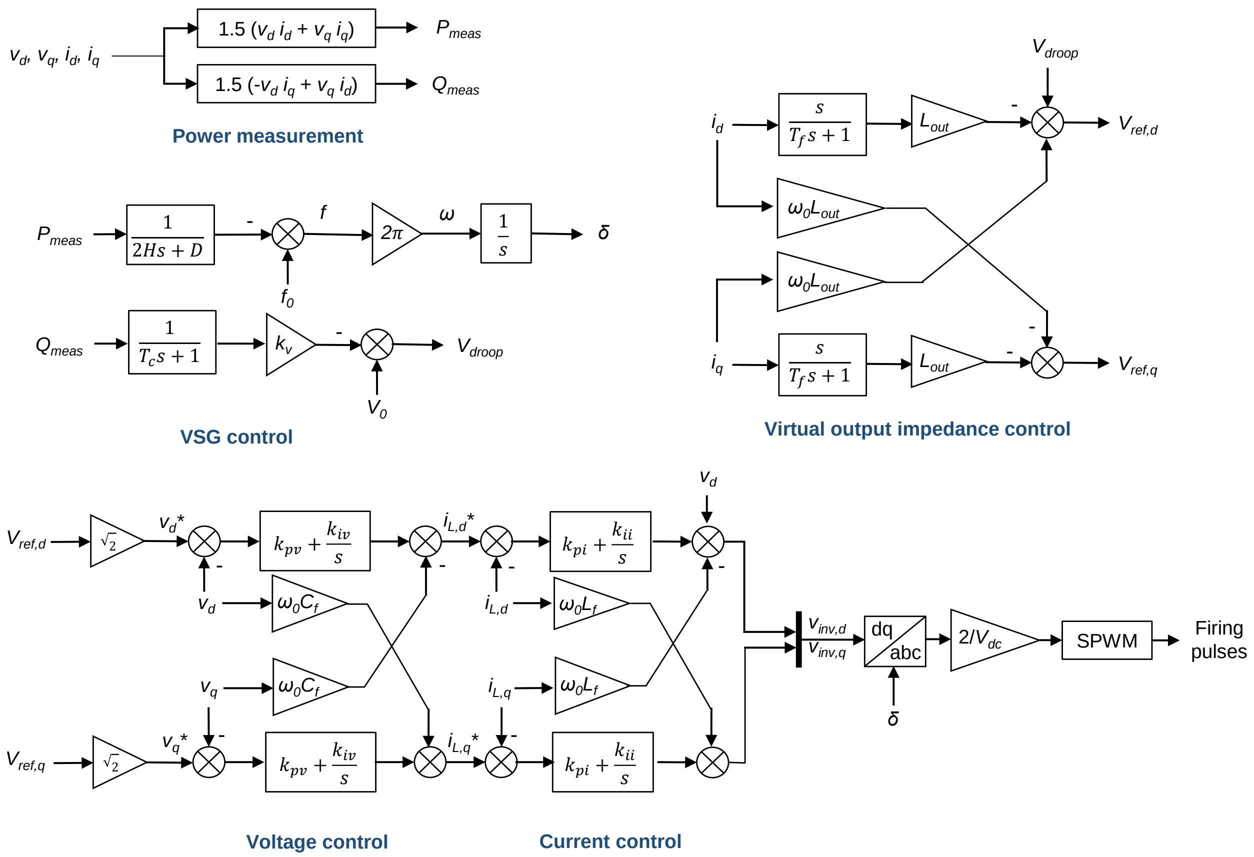


**Fig. S3.** Control block diagram for a three-phase inverter operating under VSG control and emulating a virtual output inductance $L_{out}$. Here, $L_f$ and $C_f$ are the inductance and capacitance of the output LC filter, respectively. [SPWM: Sinusoidal pulse width modulation]

# S4 Supplementary note 4: Performing small-signal stability analysis

## S4.1 Calculating the bus admittance matrix

To perform stability analysis, the bus admittance matrix $\mathbf{Y_{bus}}$ must be determined. First, to reduce the computational complexity of the analysis, we contract the network using the following steps:

1. Identify the active nodes, i.e., those with inverters or synchronous generators in the network.
2. Identify the set of nodes that are on a direct path from an inverter/synchronous generator to the root node of the corresponding subgraph.
3. Only the above set of nodes will be retained in the contracted graph. Other nodes are eliminated as follows. For all the nodes that are to be eliminated in the graph, do the following iteratively:
   (a) If a node '$b$' to be eliminated has exactly one predecessor '$a$' and successor '$c$', replace the edges $(a, b)$ and $(b, c)$ with an edge $(a, c)$ whose resistance and inductance are the sum of the resistance and inductance of the replaced edges, respectively.

Using this contracted network, the bus admittance matrix ($\mathbf{Y_{bus}}$) is subsequently calculated using the following procedure:

1. The size of the $\mathbf{Y_{bus}}$ matrix is the number of remaining nodes in the contracted graph.
2. For the $(i, j)$ element in $\mathbf{Y_{bus}}$:
   (a) If $i = j$, the matrix element is the sum of the admittances of all lines connected to that node;
   (b) If $i \neq j$ search for the lines connected directly between the $i$ and $j$ nodes and add their admittances, and reverse the overall sign.

Finally, the obtained $\mathbf{Y_{bus}}$ matrix is Kron-reduced to retain only those nodes that are active, see ref. [7] for the detailed procedure.

If a VSG contributes virtual impedance, this impedance appears in series with the inverter. On the inverter-side of this impedance, a new active node is created to represent the VSG, and the original inverter node—on the network-side of the impedance—is eliminated using Kron reduction as it becomes passive.

## S4.2 Assessing the small-signal stability of the power system

The inputs to our stability analysis are the $\mathbf{Y_{bus}}$ matrix and the droop gains of the VSGs. The poles of the system are the solutions to the following polynomial eigenvalue problem (see ref. [8] for the detailed derivation).

$$[\mathbf{A_0} + \mathbf{A_1}s + \mathbf{A_2}s^2 + \mathbf{A_3}s^3 + \mathbf{A_4}s^4] \begin{bmatrix} \Delta\delta \\ \Delta V \end{bmatrix} = \mathbf{0} \tag{3}$$

where the coefficient matrices are as follows:

$$\mathbf{A_0} = \begin{bmatrix} -(\rho^2+1)\mathbf{B} & -\rho(\rho^2+1)\mathbf{B} \\ \rho(\rho^2+1)\mathbf{B} & (\rho^2+1)(-\mathbf{B}+\mathbf{L_q}) \end{bmatrix}$$

$$\mathbf{A_1} = \begin{bmatrix} (\rho^2+1)\mathbf{L_p} & -(\rho^2+1)\frac{\mathbf{B}}{\omega_0} \\ (\rho^2+1)\frac{\mathbf{B}}{\omega_0} & ((\rho^2+1)T_c + 2\frac{\rho}{\omega_0})\mathbf{L_q} \end{bmatrix}$$

$$\mathbf{A_2} = \begin{bmatrix} ((\rho^2+1)T_c + 2\frac{\rho}{\omega_0})\mathbf{L_p} & \mathbf{0} \\ \mathbf{0} & (\frac{1}{\omega_0^2} + 2\frac{\rho T}{\omega_0})\mathbf{L_q} \end{bmatrix}$$

$$\mathbf{A_3} = \begin{bmatrix} (\frac{1}{\omega_0^2} + 2\frac{\rho T_c}{\omega_0})\mathbf{L_p} & \mathbf{0} \\ \mathbf{0} & \frac{T_c}{\omega_0^2}\mathbf{L_q} \end{bmatrix}$$

$$\mathbf{A_4} = \begin{bmatrix} \frac{T_c}{\omega_0^2}\mathbf{L_p} & \mathbf{0} \\ \mathbf{0} & \mathbf{0} \end{bmatrix}$$

where $\mathbf{L_p} = (2\pi\mathbf{K_f})^{-1}$, $\mathbf{L_q} = \mathbf{K_v}^{-1}$, $\mathbf{G} + j\mathbf{B} = \mathbf{Y_{bus}}$, $\rho$ the average R/X ratio of the lines, and $\omega_0$ the nominal frequency.

Once the poles of the system are known, the dominant mode of oscillation refers to the complex pole-pair that has the maximum real part—the damping ratio of this pole-pair is used as a measure of system stability in our study.

# S5 Supplementary note 5: Distributed stability metric

For deriving the distributed stability metric (DSM) used in our study, we refer to the scenario of a single VSG connected to an infinite bus shown in Fig. S4.

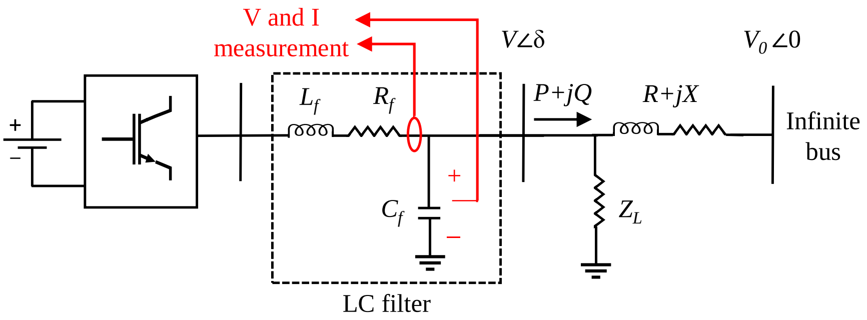


**Fig. S4.** Schematic of a VSG connected to an infinite bus through an impedance $R + jX$.

The characteristic equation for the system is as follows (see ref. [9] for the detailed derivation), considering the same terminology from the Methods section of the main article:

$$\frac{s^2}{\omega_c k_f} + \left[\frac{1}{k_f} - B' + \frac{2k_v G' G}{1 - k_v B} - \frac{k_v G^2(1 - \omega_c k_v B')}{\omega_c(1 - k_v B)^2}\right] s + \left[-B + \frac{k_v G^2}{1 - k_v B}\right] = 0. \tag{4}$$

Here,

$$G = \frac{R}{R^2 + X^2} \tag{5}$$

$$B = \frac{-X}{R^2 + X^2} \tag{6}$$

$$G' = \frac{X(X^2 - R^2)}{\omega_0(R^2 + X^2)^2} \tag{7}$$

$$B' = \frac{2X^2 R}{\omega_0(R^2 + X^2)^2} \tag{8}$$

The roots, $\lambda$, of the characteristic equation (4) are the eigenvalues of the system. Therefore, the system is stable if all eigenvalues have a negative real part. For equation (4) above, the real part of the two complex roots is the following:

$$\begin{aligned} Re(\lambda) &= -\frac{\omega_c k_f}{2}\left[\frac{1}{k_f} - B' + \frac{2k_v G' G}{1 - k_v B} - \frac{k_v G^2(1 - \omega_c k_v B')}{\omega_c(1 - k_v B)^2}\right] \\ &= -\frac{\omega_c}{2} + \frac{\omega_c k_f}{2}\left[B' - \frac{2k_v G' G}{1 - k_v B} + \frac{k_v G^2(1 - \omega_c k_v B')}{\omega_c(1 - k_v B)^2}\right]. \end{aligned} \tag{9}$$

Note that the first term in the above equation, $-\frac{\omega_c}{2}$, is a constant for an inverter. However, the second term varies with the droop gains $k_f$ and $k_v$, and the network parameters $G$ and $B$. Therefore, how close the system is to small-signal instability depends on how positive the second term is—the smaller the second term, the farther the poles are from the origin and the higher the system stability is. In contrast, if the second term is large, the poles move closer to the origin and the system becomes more unstable. Therefore, this term is chosen as the metric to quantify the stability contribution of each inverter in our study, see equation (10) below. If this metric is large for an inverter, adding the inverter to the grid will make the latter more unstable. However, instability can be avoided if the inverter participates as stability storage (see Fig. S5)—increasing the inverter's output impedance decreases the value of $B$ and hence the metric.

$$\text{DSM} = \frac{\omega_c k_f}{2}\left[B' - \frac{2k_v G' G}{1 - k_v B} + \frac{k_v G^2(1 - \omega_c k_v B')}{\omega_c(1 - k_v B)^2}\right]. \tag{10}$$

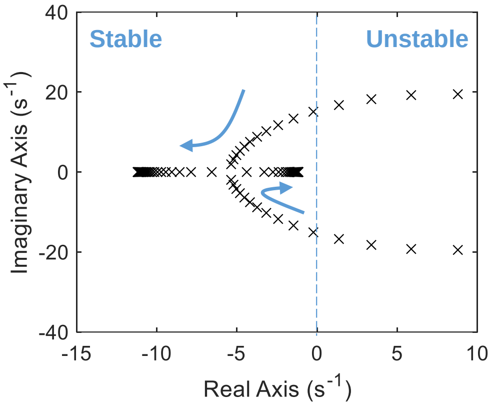


**Fig. S5.** Plot illustrating how the poles of the system become stable as the emulated output inductance increases from 0 to 2 mH for $k_f$=1.5%, $k_v$=4.5% and $R + jX$ = (0.015 + $j$0.015) pu. This corresponds to the same scenario presented in Fig. 3(b) in the main article. The blue arrows point in the direction of increasing inductance.

# References


[1] Gururaghav Raman, Bedoor AlShebli, Marcin Waniek, Talal Rahwan, and Jimmy Chih-Hsien Peng. How weaponizing disinformation can bring down a city's power grid. *PLOS ONE*, 15(8):e0236517, 2020.

[2] Gururaghav Raman, Bedoor AlShebli, Marcin Waniek, Talal Rahwan, and Jimmy Chih-Hsien Peng. How weaponizing disinformation can bring down a city's power grid: Supplementary data. `https://www.penglaboratory.com/topology-data`, 2019.

[3] Institute of Electrical and Electronics Engineers (IEEE). IEEE C37.010-2016 - IEEE Application Guide for AC High-Voltage Circuit Breakers > 1000 Vac Rated on a Symmetrical Current Basis. `https://standards.ieee.org/standard/C37_010-2016.html`, 2017.

[4] BS6346 Cables — Technical Reference. `http://www.caledonian-cables.co.uk/Product/BS6346/Technical%20Reference.html`.

[5] British Standards Institution. BS 5467:2016. `https://shop.bsigroup.com/ProductDetail/?pid=000000000030348006`, 2016.

[6] British Standards Institution. BS 6622:2007. `https://shop.bsigroup.com/ProductDetail/?pid=000000000030186876`, 2007.

[7] John J Grainger. *Power system analysis*. McGraw-Hill, 1999.

[8] Gurupraanesh Raman and Jimmy Chih-Hsien Peng. Filter deballasting control of droop-controlled inverters. *IEEE Transactions on Power Electronics*, 36(11):13107–13117, 2021.

[9] Gurupraanesh Raman, Jimmy Chih-Hsien Peng, and Hatem H Zeineldin. Optimal damping recovery scheme for droop-controlled inverter-based microgrids. *IEEE Transactions on Smart Grid*, 11(4):2805–2815, 2020.
